# Valedictory Address upon the Part of the Class, 1859, in the Atlanta Medical College

**Published:** 1860

**Authors:** B. F. Ward

**Affiliations:** Miss.


ATLANTA

### Medical and Surgical Journal.

Vol. V.] JAN'Y & FEB'Y, 1860. [Nos. 5 & 6.

### ORIGINAL COMMUNICATIONS.

### ARTICLE I.

*Valedictory Address upon the part of the Class*, 1859, *in the Atlanta Medical College.* By B. F. Ward, of Miss.

*Fellow-Students and Gentlemen of the Faculty of the Atlanta Medical College*:

It is with mingled feelings of pleasure and regret that I rise to the performance of that task assigned me by my esteemed associates and fellow-students; to act the part of their humble representative in this interview, which is fixed by the hour of separation as the last we shall hold in the respective capacities of a Faculty and a Class.

In all the domain of language, there is no word, which being the natural offspring of the soul's most generous impulses and which ever breathing the warmest spirit of love and esteem that animates the breast, yet falls so heavily from the lips as the honest adieu; no word in whose simple cadence are so completely blended the messengers of joy and regret; no word, which, while epitomizing the hidden volumes of fond attachment and fidelity of those, whose prayers for our welfare ascend as a daily incense to the God of nature, yet in its hurried utterance, throws around us such a pallor of despair, or plants in the heart such an agony of grief. Invariable in its import through all the dialects which diversify the nations of the earth, from the classic Greek to the unlettered Hottentot, it leaps not from the tongue of sincerity without spreading upon the soul of him who hears, some modified shade of its characteristic gloom.

Whenever it falls upon the ear, it comes laden with the same impressive tones, whether it be the language of tender emotion, where the pang is half soothed by the brilliancy of hope, or the bitter wails of dark and weeping despondency bending o'er the tomb of buried hopes and fallen idols.

Then, Gentlemen of the Class, in view of the relations which we have borne to each other for months just past; in view of the social attachments and the fraternizing spirit which identity of purpose and community of interest must have engendered among us; standing as we now do on the brink of separation, while the ever revolving scythe of time, descending with its constant velocity, hangs just ready to sever those mutual ties of friendly intercourse, which have so long thrown their tendrils about us and added pleasure as well as profit to the labors of our pupilage; in view of all this, would it be unnatural should there run through the proceedings of this hour, anything of that "pale cast of thought" which poets are pleased to term melancholy, but which the purest may wear without detriment to her charms, and the boldest may acknowledge without sacrifice of dignity. When visionary youth, buoyant with high hope and bold desire, tears himself for awhile away from the shrine of affection, to chase on the field of danger the fleeting form of glory, there is bitterness in the fond adieu which the tears of trembling passion speak, but sweetly neutralized by the bright anticipation which pictures his return at some distant day, laden with honors that shall challenge a prouder esteem when his devotions are renewed at his favorite altar. When the long and oft-repeated adieu of the doting parent sends the flower of his pride, in blooming boyhood, from his side, to treasure the lore of learning in the stranger's land, the cloud of sorrow which hangs over their separation, is relieved and the parental eye is cleared by that prospect which brings back, in time, his favorite boy, clothed with accomplishments which shall honor his gray hairs and cheer his declining age. The incubus of grief is lifted from the light heart of boyhood, too, by the contemplation of the happy hour when his eye shall again be greeted with the recurrence of every scene which, on the broad canvas of memory, ever clusters in wonted loveliness around his childhood's home; but when we realize in the hour of parting, the grave where must be entombed all the joys of companionship and intercourse; when hand gives back to hand the last pressure of friendship; when heart beats its last response to kindred heart, and eye lingers insatiate on the last look of love, or wanders in final view over the endearing scenes of home and nativity, then alone it is we can feel in full force the cruelty of kindness that swells the friend's adieu.

Such, Gentlemen, to some extent, is the nature of our separation to-day; yon fiery steed, all restive in his might, stands panting now in smoking eagerness, ready to measure between many of us those long miles of separation, which, perhaps, can never be abridged by all the mutations which mark the steady career of time. But we may not dwell upon this theme; let us turn from this shaded view of our relations, and glance briefly toward that mysterious future, whose sable veil no mortal hand may lift, no finite vision pierce. This hour to us is fraught with the deepest interest; it must form an era of no little consequence in the history of each individual concerned, since it puts a new phase upon the course of his life; plants him on a new and extended field of action; changes his relations to those under whose guidance and by whose aid he has reached his present position; gives him new relations to society at large and to those who may look for benefaction to his acquirements and skill; and imposes upon him responsibilities numerous and grave, and which it requires courage and confidence to meet, vigor and energy to bear; these responsibilities he must to day assume, and if he feel not the weight of their assumption, he is stultified; if he regard them not, he is base; if he shrink from them, he is craven. But what are the directions of these responsibilities? He is responsible, first, to his own sense of rectitude, in entering upon the course which from this hour lies before him. He must answer to conscience, stern and inexorable judge, as to whether he has prepared for the great work which henceforth devolves upon him; even here, ere his armor is buckled on, the inquiry comes up to his intelligence and honesty, whether he has provided for the contest that awaits him. This, Gentlemen, is a momentous inquiry; this appeal, which is ever welling up from the living springs of humanity and addressing itself to the conscience of him who deals with the life of his fellow.

This is the primary, the silent but deeply impressive interrogation which claims the consideration, ah! and the affirmative response of every soldier who draws an unfleshed sword beneath the Esculapian banner. The mail-clad hero who tilts for the victor's prize, enters not the tournament till his visor is closed, his shield burnished, and his lance well chosen; and, yet, with fair precaution, and timely warned by a generous rival, he may fall like the proud Templar, before the unerring aim of the "disinherited Knight." The wary chieftain who leads his van in the tide of victory, goes not to the charge till the fire of conflict burns in every breast; till every heart is steeled with fury, and the dark defiance which gleams from the eye writes victory on every brow, and then sweeps on, like an avalanche, to triumph, because there is prisoned in every bosom that spirit which gives consciousness of strength. But he who goes out to war with disease, must meet an enemy more fierce than the bearded foe that buried in "Byzantium's tomb" the last of Grecian glory; more potent than "the red arm of Goth and Vandal" that waved the sword of contest over the shrines and palaces of imperial Rome; more artful and insidious than the cruel savage who lurks beneath the grass on the way-side of his victim. Yes, more obstinate, more formidable is this enemy of health and life, than all the engines of war that dip their lips in human gore. Already, too, he awaits your coming on the plains of combat, arrayed in all his native terrors; his pallid hand pointing to the cheek of health and beauty; his blighting breath steaming from his pestilential nostrils, while from his dark pinions, he shakes misery and death; his feverish arm encircles the tottering frame of hoary age; the stalwart powers of vigorous manhood, whose struggles in his grasp are fierce as the throes of expiring liberty; and the tender form of budding loveliness, upon whose brow sit the cold dews of death, "like an untimely frost upon the sweetest flower of the field"; the wails of desolation and grief come up from his dominions, like the sad requiems from the houses of the first born of Egypt. Wherever the eye may turn, ten thousand bleaching monuments of his might heave up their sepulchral forms, while his millions sleep unchronicled in the Mausoleum of ages.

Not only does he spread havoc and destruction in his way, as he comes robed in these habiliments of terror; but old Proteus like, he makes his descent in every garb which the elements of nature can furnish; he may come in murky cloud, from the foulest seething caldron of decaying nature; he may float on the balmiest breeze that fans the burning brow; ride on the modest sunbeam or genial shower that comes to wake the energies of earth; or spring on vapory wings from the silvery dews that bead the tapestry of nature, when "cheerful morn, all sweet and fresh, spreads from the rosy east;" he may spring from the bubbling fountain, the floral fields, the mirroring lake or the blue waves of Neptune's deep domain; from the little cryptogam which is born unseen on the very atmosphere of life; indeed, from every crevice in nature's vast arcana, this messenger of pain hurls his deadly missile or lifts, in disguise, his poisoned chalice to the lips of man. Who, then, can meet his challenge, which is thrown to every disciple of the healing art, unless he can respond to this first great inquiry, that he has fairly donned the helmet of science; laid broad and deep the foundation on which to rear his barracks of defence; entered fully into the premises of his foe; laid bare his plan of operation; exposed his modes and points of attack; acquired his history; investigated the type and features of his character, and clearly mapped out every lineament of his metamorphic phasis? True, he may hold in his hand the only credentials which the proudest Institution and the noblest Faculty in the land can grant; but these, without the self-reliance which nought but the innate consciousness of strength can give, is frail as the autumnal leaf before the gathering storm.

The Physician's next responsibility is to his fellow-beings, and especially to those whose health, whose happiness and life may, by the influence of fortune or merit, be suspended on his capacity to discharge these duties which he has voluntarily and publicly assumed; those upon whose arms he is borne up in his professional capacity; those who hail him as a ministering angel; as a messenger of peace and comfort when

> "Dire disease, whose ruthless power
> Withers the beauty's transient flower,"

has swept through the halls of mirth and left pale sorrow weeping by the hearthstone; those who invite him beyond the vestibule of the sacred circle and make his own bosom the safe repository of priceless confidence; who give their fidelity to the honesty of his assumptions, and place their reliance on the verity of his passport and the value of his claims; here is a tacit but mutual compact which should be strong as the golden strands of young love's plighted vow, and inviolable as the Heaven-sealed oath.

Can the Doctor of Medicine enter upon the duties of his office and avoid the weight of this silent obligation? He cannot. The very instrument with which his fostering parent sends him on his mission, proclaims his willingness, his desire and his ability to meet the demands of those claims which suffering humanity holds upon the votaries of his calling.

But the duties which the physician owes to society at large, embraces a wider field than this; it is not only his privilege, but it is properly incumbent upon him to stand committed to the support and furtherance of every enterprise or design which has for its object the dissemination of knowledge, the exposition of error and the elevation of the moral standard. Not only as a philanthropist should he look out with interest on the great strife which burns between the powers of light and darkness; not only as desiring to promote the general good of mankind, should he ever be ready to throw the whole weight of his influence with truth and virtue against the allied forces of vice and error; but with the view also of securing, to his own time-honored calling, that respect and deferential consideration which are ever awarded to it by the wise, the enlightened and refined; but which it can never enjoy while immersed in the enervating gloom of ignorance and the dark train of evils which multiply and grow in its sickly atmosphere.

The next obligation which every medical man should feel, is to support the character and dignity of his profession. We enter to-day, gentlemen, the portals of a profession which, in its antiquity, its beauties, its magnitude and grandeur and the sublimity of its purposes, is unsurpassed by any of its proud sisters that shine in the great galaxy of sciences.

No science has advanced more rapidly and steadily to perfection since the day when the great Disciple of "Cos" carried his innovations into the rudimentary doctrines of the "Asclepiades," stirring afresh the spirit of research and investigation and communicating a new impulse to the wheels of progress. Who does not feel the kindlings of a new enthiasm, the impulse of prouder emotions, as he finds himself fairly enlisted on that field of noble service, which has so long fructified under the life-giving labors of the mightiest intellects; that field now teeming, in rich profusion, with the gems of truth which the genius of other ages has shed upon it?

What mind stands not in mute admiration and wonder, before the ancient grandeur of that colossal temple of Medical Science, around whose broad base the circling waves of twenty centuries have rolled; planting, as it now does, its time-stained pedestal on the two great continents of earth, and stretching away, in its vastness, to the land of Pyramids and the kingdoms of the Orient; arching, in gothic greatness, over the world of waters, from the golden gates of morning to where the "proud Pacific chafes her strand;" consecrated for all time by the memory of its illustrious architects; of Hipocrates, Galen, Celsus and Pare; Hunter, Harvey and Monroe, and all the innumerable host of the immortalized whose names rise in brilliant succession along the registry of this vaulting immensity, from the foundation segment inscribed with the name and fame of the old "Parens Medicinæ," to its towering summit now blazing with a brighter reflection from the lustre of Minerva's shield, and about which clamber, in green festoons, the garlands of modern champions? But it is not this view of the modern and antique majesties of the Medical Profession, which should exert the deepest veneration of its beneficiaries; which should most excite the honest pride and elicit the noblest service of those who aspire to the honors of its mantle; but it is its humane and laudable purpose; the sacred commission which it confers on those who go out to do its behests.

No scientific profession garners a richer store of benevolence than this; none dispenses a wider fruition of charities, those ripe fruits of human kindness whose quality "is not strained, but droppeth as the dews from Heaven."

> "Unfee'd the calls of nature she obeys,
> Not led by profit nor allured by praise."

Wherever the cry of pain is heard, or the wasting of disease is felt, her messengers are found; whether it be in the palace of splendor, where the rioting child of fortune groans upon his couch of luxury, or in the pest breeding hovel where "sickness sits caverned in the hollow eye" of poverty and degradation; under all circumstances, in all seasons and at all hours, whether invigorated and sustained by all the ardency of hope and the smiles of prosperity, or intimidated by the frowns of adversity, oppressed and born down by the leaden weights of care and grief; whether it be when merry spring crowns with smiling verdure the youthful year and sheds gladness upon the earth; or beneath the sultry summer's sun, when the hurricane of disease, borne upon miasmatic wings, bears down, like a scorching Simoon, upon the land; or when frigid winter clanks about the earth his icy chain and shakes his dreary locks in the face of shivering misery; whether it be in the light and comfort of day, or in the still, dark hours when the sable goddess stretches over a slumbering world her leaden sceptre, and nature, worn and wearied, longs for the arms of her sweet restorer; this slave to the ills of flesh, who is pointed at by poor, driveling, brainless ingratitude as a heartless, mercenary beast fattening upon the afflictions of his neighbor, must go with energy and promptitude, on his toilsome round of duty, while his stupid vilifier is wrapped in the narcotizing slumbers of a weak and groveling mind. More than this, it is the province of this profession to play an active and efficient part in the execution of the most exalted aims of government and law; to give a strong hand to the protection of innocence; a fierce and sleepless eye to the pursuit and detection of guilt; to extend a gigantic arm over that "reputation" which, being lost, "all that is left is bestial;" to sit in judgment upon character when the fidelity of friends is shaken by the "green-eyed monster" of suspicion, and rescue or ruin hangs pendulous upon the fiat of the medical man. No avocation places its votaries on higher ground or sets before them stronger motives to strike for eminence in rank; none demands, as the price of distinction, a sounder information or a higer degree of mental and moral culture.

Beyond its peculiar sphere of duty, it is immediately encompassed with the great theatre of real life, whose ample and checkered stage is ever inviting into action all the higher attributes and nobler qualities of man's nature.

The medical man is the companion and confidant of all casts and grades of character; he treads alike the great highways of learning and religion, and the dark and loathesome avenues where lawless debauchery holds her midnight orgies, and haggard want writhes in the agonies of untimely disease; duty carries him into the strongholds of iniquity and to the cold, dark cell, where the mildews of desolation have gathered upon the threshold of despised merit and perishing honesty; he is called to expend his skill on the very hot-bed where vice is born and nurtured, and where the Siren tongue of temptation chants, with fatal melody, the measures of her lay; he finds the neglected haunts where the warning and vitalizing voice of instruction resounds not; where the hand of charity is withheld and the cornucopia of benevolence is never turned.

But while all this is true of the Medical Profession as a body, we have to acknowledge a long and parasitical appendage, composed of those zoophytes of the human family who, content to live and thrive upon the succulence of a name, cling with fungous tenacity to that great mass of which they cannot form a part; those mongers, and charlatans, and imbecile pretenders who are supposed, by the casual observer, to have degraded a noble cause, whose surface they merely dot as foul excrescences; and who go forth to the world with falsehood written upon their foreheads and double-edged daggers in their hands.

Who, then, in taking upon himself that title which he is adjudged as worthy of bearing, shall be insensible to the obligation which requires him to so develope, in his own course of action, the true features of the cause which he has espoused, as to aid in eradicating that wide spread misconception which would allot to the Medical Profession that refuse of talent which is denied a seat in the halls of Theology and Law?

How many sad evidences have we of the existence and prevalence of this morbid sentiment! The mis-guided parent seeing, in the mental manifestations of his son, no index of those powers which might do honor to the Bar or the Sacred Desk, plant a flower in the gardens of literature or lift an audible voice in the councils of the nation, is prone to seek relief for the shaded and monotonous picture of his child's destiny in the hope, ah! delusive hope, that he may still make a Doctor of him. Oh! miserable error! oh! ruinous deception! why should the flickering intellect, which is unable to turn a broad disc of illumination on the truths of the simplest science, be expected to light the ample and multiplied chambers to which lead the deep and torturous labyrinths of Medical Philosophy? that science which girdles the world in its circuit and levies its contributions upon every department of nature; that seven-fold science, either one of whose great radicles, in its countless ramifications, would employ for an age, the labors of the most Herculean mind; and yet, the crippled intellect, whose feeble and unfledged powers of reason can scarcely combine the elements of the simplest syllogism, is required to reign over these united dominions whence medicine draws her resources, and to guide, regulate and control the movements of this "fearfully and wonderfully made" complexity, the animal economy, whose beauties he can never see; whose mysteries he will never know, surrounded, as he is, with a "*nigra zona inscientæ.*"

Another responsibility to which the young Physician should be fully alive, is that to which he owes to his "Alma Mater;" to the Institution under the light of whose countenance he goes out to claim his place in the ranks of his brethren; that Institution which not only his love of learning, his regard for the wants of his country and the cause of humanity, but which an honest pride and self interest call upon him to honor and support by every power of his mind, every energy of will and every virtue of his character.

We can to-day, gentlemen, realize the full force of this obligation! We go forth as the largest graduating class of a young but noble and rising Institution, which, however dark may have been the period of its infancy; with whatever adversities it may have struggled in times that are past, has shaken off the drapery of ill-foreboding, risen above the dust and smoke of conflict and reared its proud dome into an unclouded sky, where it is kissed by the rays of an auspicious sun, and gives back the music of gentle zephyrs which tell of future greatness. Already has her deep, clear voice rolled away to the Eden of the land, the Valley of the West, and died in echo beyond where the "king of flood" rolls his turbid waters. Southward and Westward has she moved with majestic stride, stretching her fair hands to her elder sisters; not subordinately, but as a princess of the blood, bringing grace, strength and beauty to the grand alliance.

Medical Colleges, in one sense, are the great hearts of the nation, whose annual pulsations send out, in every direction to the vast tissue of society, the elements of their own elaboration. So long as these elements are sound and healthful and highly wrought, they carry life, vigor and durability to the great structures they supply; but if they are crude and noxious and non-vitalized, then must the mighty organism to which they are dispensed, receive into its meshes that which shall "touch corruptibly the life of all its blood" and breed disease, degeneration and decay. This class, Gentlemen, is the elaborated material just issuing from the laboring recesses of our own vigorous organ. Shall it not be the living resolve of every man that no stain shall flow back to its portals through the channel of his career? Be it so; may every man so run the race which is set before him, that he may add new sinew to her arms, new symmetry to her form and new confirmation to the great principles she avows and teaches. Let each and all of her offspring live up to the injunction she lays upon them and prove themselves worthy of their heritage, that the "Atlanta Medical College" may enjoy, in the deeds and virtues of her own posterity, a life that shall but climb to its zenith, when that corroding breath of changes, which stamps mutability on all human grandeur" and crumbles into dust the noble monuments of art and genius, shall have swept her strong foundations from the earth, and the weeds of desolation alone mark the spot where she stood.

And to you, Honored Sirs, whose high privilege it is to guard her interests and watch over her destiny, to you we feel bound by every consideration, which establishes our allegiance to that Institution of which you are the guardian spirits, and at whose altars your offerings are so devoutly made; you whose province it has been to shed the light of reason and experience on the mysteries of that great volume of nature whose pages are spread for our perusal; and to lay the strong hand of science on those frowning fallacies which ever rear their wrinkled fronts to impede the march of truth.

From you, Sirs, and your halls of instruction we go to cherish and esteem that which shall but grow and ripen with the full development of those principles you have given us and exhorted us to nurture and preserve. Long may you live to carry forward the great work which is upon your hands, and when a green old age shall have called you from your labors with all the honors of the faithful, the valiant and the strong, clustering upon your brows, may your eyes be gladdened with the happy prospect of the seed, which you have sown, rising with pillared strength to catch up and bear on, without blemish, the fair folds of your drooping mantle.

---

*Ladies and Gentlemen, Citizens of Atlanta—*

For the array of beauty, intelligence and dignity which you have brought to our Commencement, and for the interest which you added to the occasion, permit me, on my own part and in behalf of those for whom I have the honor to speak, to tender you our warmest gratitude, and bid you a heart-felt adieu. Your hospitalities, your courtesies, your refinement and christian character, have given you a warm place in the hearts of the Class now before you; and the associations we have enjoyed among you will form living jewels in the casket of memory, about which the kindest reflections will gather when to-day and its scenes shall have long been numbered among the triumphs of time.

We do earnestly hope that succeeding years may never bring to your homes a Medical Class, who shall abuse or fail to appreciate the generous welcome you are wont to extend them; and when your infant city, which, even now, lays one hand on the "Father of Waters" and the other upon the Ocean's cheek, shall have attained that magnitude and distinction to which she is so early destined; with her growth of commerce, her increase of wealth and power, and her uprising of education and virtue, may there also be an increase of her interest in the prosperity, the permanence and renown of the Atlanta Medical College.

